# Ligand-Target Prediction by Structural Network Biology Using nAnnoLyze

**DOI:** 10.1371/journal.pcbi.1004157

**Published:** 2015-03-27

**Authors:** Francisco Martínez-Jiménez, Marc A. Marti-Renom

**Affiliations:** 1 Genome Biology Group, Centre Nacional d’Aanàlisi Genòmica (CNAG), Barcelona, Spain; 2 Gene Regulation, Stem Cells and Cancer Program, Centre for Genomic Regulation (CRG), Barcelona, Spain; 3 Institució Catalana de Recerca i Estudis Avançats (ICREA), Barcelona, Spain; Icahn School of Medicine at Mount Sinai, UNITED STATES

## Abstract

Target identification is essential for drug design, drug-drug interaction prediction, dosage adjustment and side effect anticipation. Specifically, the knowledge of structural details is essential for understanding the mode of action of a compound on a target protein. Here, we present nAnnoLyze, a method for target identification that relies on the hypothesis that structurally similar binding sites bind similar ligands. nAnnoLyze integrates structural information into a bipartite network of interactions and similarities to predict structurally detailed compound-protein interactions at proteome scale. The method was benchmarked on a dataset of 6,282 pairs of known interacting ligand-target pairs reaching a 0.96 of area under the Receiver Operating Characteristic curve (AUC) when using the drug names as an input feature for the classifier, and a 0.70 of AUC for “anonymous” compounds or compounds not present in the training set. nAnnoLyze resulted in higher accuracies than its predecessor, AnnoLyze. We applied the method to predict interactions for all the compounds in the DrugBank database with each human protein structure and provide examples of target identification for known drugs against human diseases. The accuracy and applicability of our method to any compound indicate that a comparative docking approach such as nAnnoLyze enables large-scale annotation and analysis of compound–protein interactions and thus may benefit drug development.

## Author Summary

Description of the “mode-of-action” of a small chemical compound against a protein target is essential for the drug discovery process. Such description relies on three main steps: i) the identification of the target protein within the thousands of proteins in an organism, ii) the localization of the binding interaction site in the identified target protein, and iii) the molecular characterization of the compound’s binding mode in the binding site of the target protein. Here, we introduce a new computational method, called nAnnoLyze, which uses graph theory principles to relate compounds and target proteins based on comparative principles. nAnnoLyze aims at addressing two of the three previous steps, that is, target identification and binding site localization. Our results suggest that the nAnnoLyze accuracy and proteome-wide applicability enables the large-scale annotation and analysis of compound–protein interaction and thus may benefit drug development.

## Introduction

The number of newly approved drugs has been significantly decreasing over the last two decades [1]. To make things worse, the therapeutic dogma that has prevailed over the years aimed at single target-specific ‘magic bullets’ against each disease. However, proteins act in complex interconnected networks, and thus, this ‘one gene, one drug, one disease’ paradigm is now clearly challenged [2,3]. The polypharmacology concept, which relies on the fact that a drug can modulate its activity by interacting with multiple targets rather than just one, was proposed to address these limitations [2]. Polypharmacology is especially valid in complex diseases like cancer or central nervous system disorders where the modulation of the activity of one single protein is not sufficient to obtain a therapeutic effect [4–6]. Therefore, identification of all possible targets of a chemical compound is critical in the drug discovery process.

Many *in silico* methods have been published for drug target identification using network approaches [7,8]. Broadly, we can distinguish two different classes of methods, structure-free methods and structure-based methods. Within the first group, there are methods based on ligand features [9] that have been successfully used to identify numerous experimentally validated interactions. However, they have difficulties in identifying interactions for drugs with novel scaffolds [10] or for targets with no bioactivity information. Others, named network-based approaches, exploit network properties to provide the drug target interactions and drug repositioning opportunities [11–18]. Although the accuracy of predictions by these methods has significantly increased, the majority cannot explain the mode of action of the drug over the predicted target due to the lack of three-dimensional (3D) information about the ligand and/or the target. The use of 3D structural data helps addressing such limitation. The most popular structured-based methods rely on molecular docking approaches performing a virtual screening of a compound against a limited number of protein targets or of several compounds against one protein target [19–21]. As a result, they provide structurally detailed information about the likely interaction between the compound and its target/s. However, the computational requirements of such approaches make them not generally applicable at proteomic scales. An exception to this limitation is the recent massive human screening of 600,000 drugs against 7,000 human protein pockets by Cardozo and colleagues whose results are available online [22]. To overcome the computational limitations, new structure-based methods use the so-called “comparative docking” approaches that solely rely on structural comparisons, both of compounds and protein targets, to infer new interactions [23,24]. Other methods use local structural comparisons of small molecule binding sites to infer the localization and specificity of binding pockets [25,26] as well as to infer new ligand interactions in known binding pockets [27]. Finally, several other methods that rely on 3D structure comparisons that aim at functionally annotating structures [23,24,28].

Here we introduce nAnnoLyze, a network-based version of the comparative docking method AnnoLyze [23]. Our new method predicts interactions for any query compound against an entire 3D proteome by relying on a bi-partite network of interactions and similarities. Unlike Annolyze, nAnnolyze can predict interactions for any compound regardless if they have been previously co-crystallized with a protein. We have benchmarked nAnnoLyze against a dataset composed by all the interactions for approved drugs present in the Protein Data Bank (PDB) [29]. The method outperforms AnnoLyze precision by 27 folds. Both Annolyze and nAnnolyze have been already successfully applied. Annolyze was used in an open source drug discovery initiative against neglected tropical diseases [30] while nAnnoLyze has been applied to a set of anti-tubercular drugs against the *Mycobacterium tuberculosis* proteome [31]. Here, we describe the method alongside the predictions for all the small molecule drugs present in DrugBank [32] against the human 3D proteome. To our knowledge, this is the first screening of almost 6,000 drugs against the entire human structure proteome predicted by comparative approaches. The nAnnoLyze network, method and predictions are available online at http://www.marciuslab.org/services/nAnnoLyze.

## Results

### Benchmark dataset creation

The correct selection of a benchmark dataset is one of the most important steps in assessing the accuracy of a newly developed method. Unfortunately, there were no available and adequate datasets for benchmarking structure-based network methods for ligand-target prediction. The “Yamanishi-2008” dataset [11], which has widely been used previously, could not be used here due to the limited structural coverage of its targets, which added to the increasing concern on biases of the current drug-target interaction datasets [33]. To address these issues, we have generated a benchmark set consisting of a “positive” and a “negative” set. The “positive” set contains all drug-protein annotated pairs between any structure in the PDB and any compound approved by the FDA. The “positive” benchmark set resulted in a total of 6,282 interactions and is considered the “true” set of interactions. The “negative” set was generated by randomly selecting pairs of compounds and targets that have never been annotated in the DrugBank or PDB databases. To assess how many of these drug-protein negative pairs could result as a potentially miss-annotated negative interactions we looked for similar compounds interacting with the “negative” target of each compound. The search resulted in 118 (∼2%) out of the 5,981 pairs that could result in a miss-annotated negative interaction. However, the removal of these pairs of putative miss-annotated “negative pairs” from the set had no effect on the assessment of the nAnnoLyze accuracy. Our final benchmark dataset included thus a total of 6,282 drug-target in the “positive” interactions and 5,981 negative pairs.

### nAnnoLyze benchmarking

**Fig 1:**
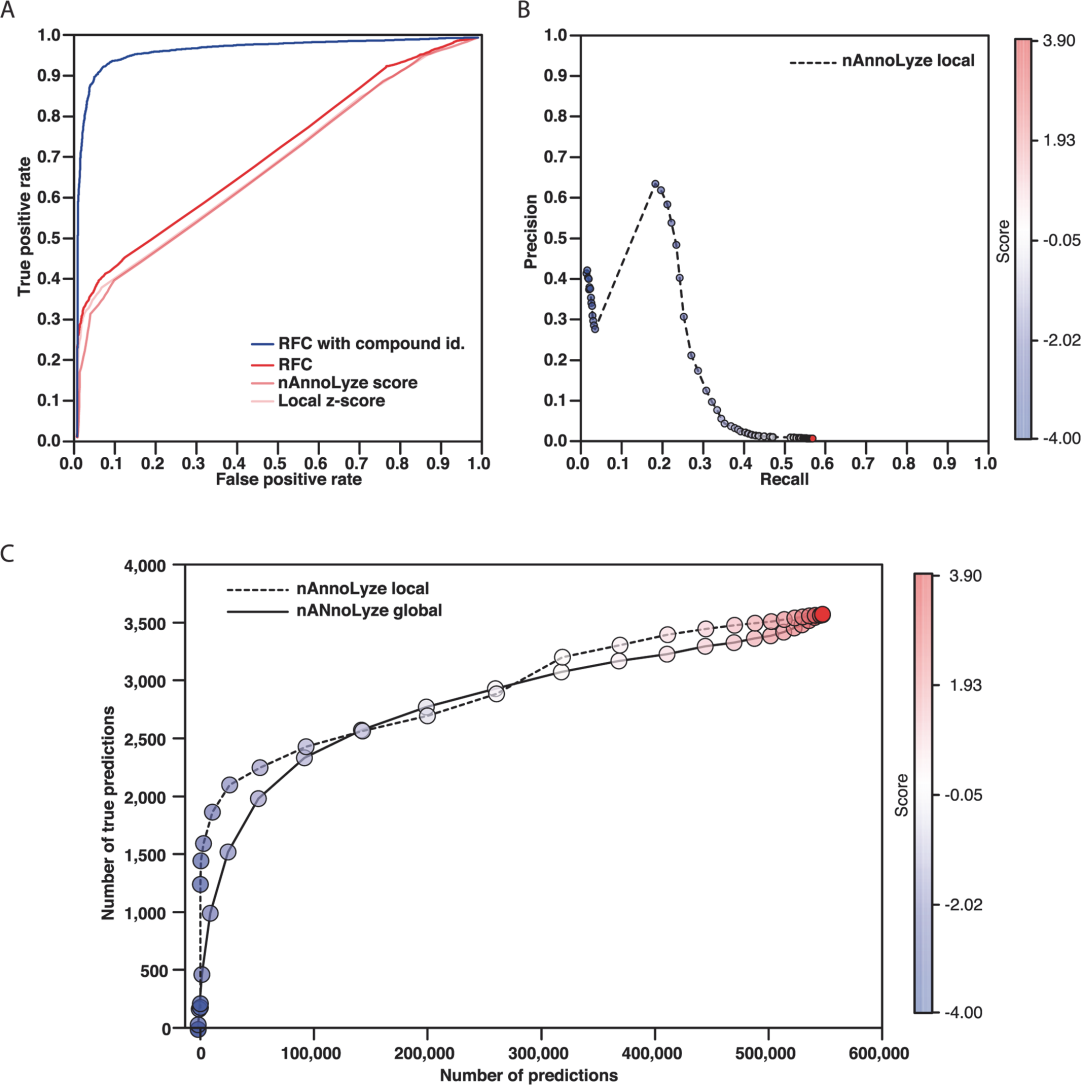
nAnnoLyze benchmarking. A) ROC plots for predictions in the benchmark dataset using 10-fold cross-validation. Blue line for predictions based on our RFC trained using the compound ID, red line for predictions based on our RFC trained with anonymous compounds. Light red lines correspond to the predictions based on individual nAnnoLyze scores. B) Precision/Recall curves for nAnnoLyze local Z-score. C) Enrichment plots for nAnnoLyze local Z-score (dashed black line) nAnnoLyze global Z-Score (solid black line) predictions.

The nAnnoLyze precision varies at different Z-score thresholds (Fig. 1A) with an optimal threshold at −2.5 local Z-score resulting in a precision of 0.63 and coverage of 0.19 corresponding to 1,148 true positive predictions (Fig. 1B). It is important to note that both the precision and coverage of our method depend dramatically on the definition of false positives for our predictions. Given that our benchmark set relies only on deposited data in the PDB, many of the predictions by nAnnoLyze are likely to be correct despite not being present in our benchmark. For example, the drug Enalapril (DB00584 DrugBank identifier) has been co-solved in only two PDB entries (*i*.*e*., 2X90 and 1UZE). However, nAnnoLyze predicts interactions between Enalapril and three other targets in the PDB (*i*.*e*., 2X91, 1J36 and 2X8Z). Those structures actually correspond to the same target sequence (Q10714 UniProt id) being solved with no ligands.

To further increase the accuracy of our predictions, we implemented a Random Forest Classifier (RFC) that classifies pairs of compound-protein as binders or not by combining several of the nAnnoLyze scores (that is, the raw score, the Local Z-score, and the Global Z-score). The RFC correctly recalled 66% of the pairs with a precision of 0.73 and an AUC of 0.71 using a 10-fold cross validation (Table 1). The tested RFC did not include the DrugBank ID as input feature to simulate a situation where a completely new compound not deposited in the databases was tested. However, by using the DrugBank ID as an input feature, the accuracy of nAnnoLyze dramatically improves to a 0.93 precision, 0.93 recall and a 0.97 of AUC (Fig. 1A and Table 1). These results suggest that predictions for known drugs already in our dataset are much more precise than those for unknown or anonymous compounds. The RFC outperformed the use of any of the single scores from nAnnoLyze (Table 1).

**Table 1:** RFC benchmark.

Type of classification	Precision	Recall	AUC
**RFC (DrugID, SCORE, Global Z-score, Local Z-Score)**	0.93±0.01	0.93±0.01	0.97±0.01
**RFC (SCORE, Global Z-score, Local Z-Score)**	0.73±0.01	0.66±0.01	0.71±0.01
**Score**	0.70±0.02	0.64±0.02	0.68±0.02
**Global Z-score**	0.70±0.02	0.64±0.02	0.68±0.02
**Local Z-score**	0.73±0.02	0.64±0.02	0.67±0.01

Mean values and standard deviation after 10-fold cross-validation.

Comparatively, nAnnoLyze reached a 0.61 increase in precision at the optimal cut-off (from 0.02 to 0.63) at the expenses of a decrease in recall by 0.38 with respect to AnnoLyze (Table 2). Finally, It is important to note that the benchmark set used for this test resulted more difficult for AnnoLyze than the original test-set used to benchmark it [23] (Table 2).

**Table 2:** nAnnoLyze benchmark.

		optimal cut-off (max value)
**nAnnoLyze**	Precision	0.63 (1.00)
	Recall	0.19 (0.59)
**AnnoLyze**	Precision	0.02 (0.06)
	Recall	0.57 (0.67)

### nAnnoLyze prediction examples

**Fig 2:**
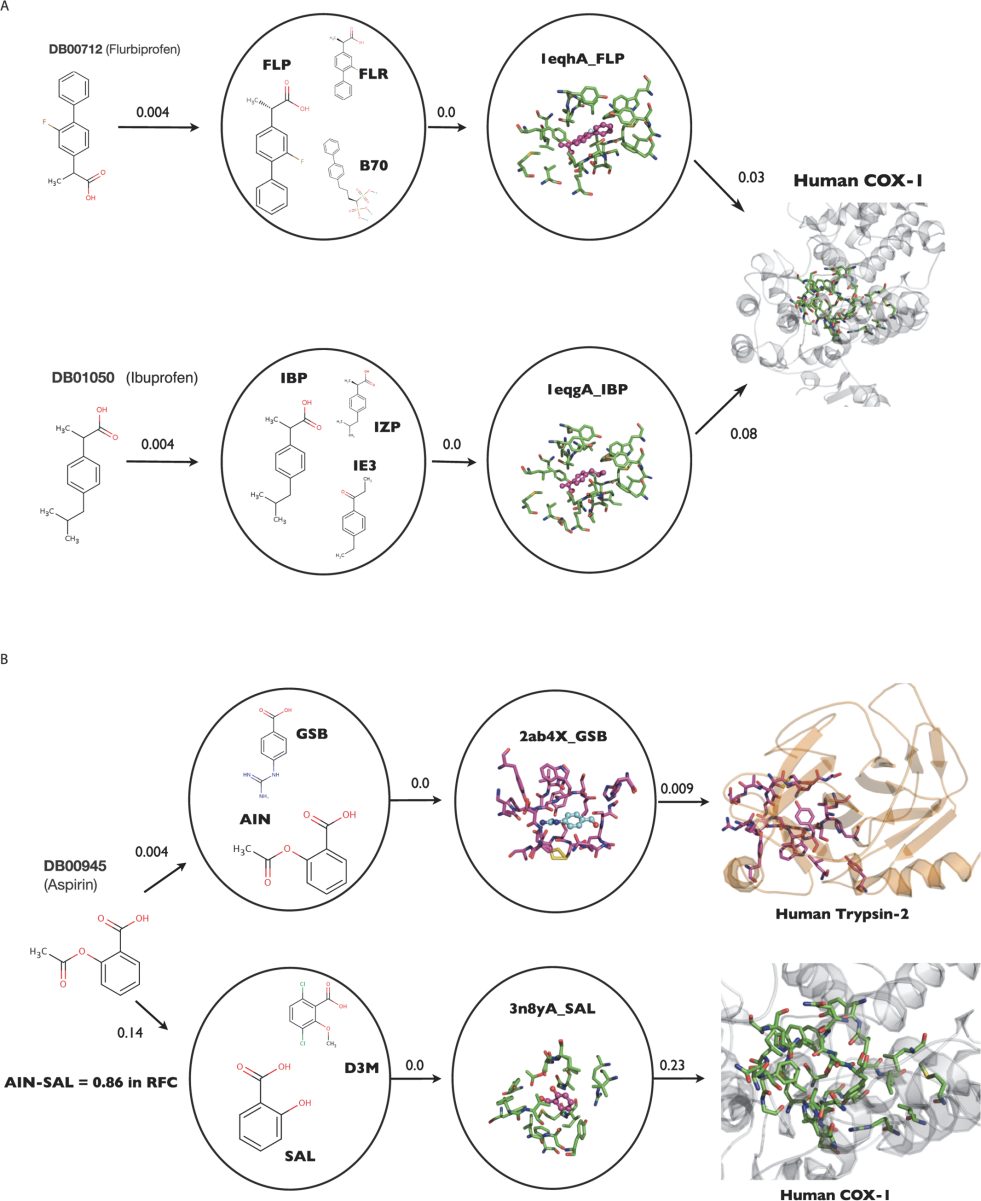
nAnnoLyze network pathways. A) Network pathways for the predicted interactions between Flurbiprofen and Ibuprofen with the generated three-dimensional model of the Human Cox-1 (PDB Template: 2AYL with 94% sequence identity). B) Aspirin network pathway for miss predicted Aspirin-Trypsin2 (PDB Template: 3P95 with 98% Sequence Identity) and correctly predicted Aspirin-COX1 hit found by the method. Link between Aspirin and SAL was made by the RFC classifier with a score of 0.86 (Tanimoto value of 0.76). In both panels, the arrows represent edges with their weights representing the distance (i.e: the inverse of the similarity). The higher the distance value the lower the similarity between the compounds or binding sites. Ligand network nodes are encircled with the ligand responsible of the predicted pathway represented in larger size. For clarity, only one binding site per node has been plotted.

The human Cyclooxygenase-1 is targeted by NSAID drugs. Cyclooxygenase (COX) is the enzyme responsible for the formation of prostanoids, which are classified in 3 different groups: prostaglandins, prostacyclins, and thromboxanes, each of them is involved in the inflammatory response, among other processes. There are two COX isoenzymes. COX-1 promotes the production of the natural mucus that protects the inner stomach lining while COX-2, is primarily present at sites of inflammation [34]. Traditional non-steroidal anti-inflammatory drugs (NSAIDs) such as Aspirin, Ibuprofen or Flurbiprofen are considered non-selective because they inhibit both COX-1 and COX-2. The inhibition of COX-2 by NSAIDs results in the anti-inflammatory effect, while the inhibition of COX-1 can lead the undesired side effects such as damage to the gastrointestinal tract [35]. nAnnoLyze predicted interactions for several NSAIDs with the 3D model of the human COX-1. Specifically, nAnnoLyze predicted 21 (out of the 44 approved drugs against COX-1) as binders of the COX-1 target (Table 3). In particular, nAnnoLyze predicted the binding of Flurbiprofen (DB00712) and Ibuprofen (DB01050) to COX-1, which are known inhibitory drugs of the human COX-1 (Fig. 2A). The nAnnoLyze path between Flubiprofen and COX-1 starts from a ligand node composed by tripotassium (1R)-4-biphenyl-4-yl-1-phosphonatobutane-1-sulfonate (B70) and two stereoisomers of Flubiprofen (FLR and FLP). Thorough the binding site of FLP to ovine COX-1 (1QEH), nAnnoLyze predicts its binding site of the COX-1 human 3D model. Conversely, the path between Ibuprofen and COX-1 starts in the ligand node composed by 1-(4-ethylphenyl)propan-1-one (I3E) and two stereoisomers of Ibuprofen (IBP and IZP). Those ligands are predicted to bind the same predicted binding site of the human COX-1 thanks to its similarity to the crystal structure of the ovine COX-1 (1EQG). Remarkably, the human COX-1 predicted binding site includes the tyrosine 385, which is known to be responsible of the catalytic reaction with the NSAID drugs (Fig. 2A). However, not all the NSAIDs performed with the same accuracy. Aspirin (DB00945), also a known inhibitor of the human COX-1 and COX-2, results in false positive predictions (Table 4 and Fig. 2B). The nAnnoLyze search with Aspirin as input molecule results in many proteases predicted targets. This false-positive pathway starts from the ligand node composed by two Benzoic Acids, the 4-Guanidinobenzoic Acid (GBS) and the Acetylsalicylic acid (AIN). GBS has been crystallized with different trypsin proteins so the pathway goes thorough the GSB binding site of the guanidinobenzoyl-trypsin acyl-enzyme (2AH4) reaching eventually the predicted binding site for the human Trypsin-2 (P07478). The same pathway is used to find other proteases like the Airway trypsin-like protease 4 (Q6ZWK6) or the Trypsin-3 (P35030) resulting in several false positive predictions. Conversely, the Aspirin-COX1 network pathway starts from the ligand node composed by 3,6-dichloro-2-methoxy-benzoic acid (D3M) and Salicylic acid (SAL) (Fig. 2B). The RFC classifier identified a network link between Aspirin and the SAL compound with a similarity score of 0.86. This SAL mediated pathway guides the nAnnoLyze search towards its binding site in the ovine COX-1 (3N8Y), which is homologous to the human COX-1 binding site. This pathway is also the responsible of the link between Aspirin and the human COX-2 with a score of 0.77. However, the lower similarity between the predicted human COX-2 binding site and the ovine COX-1 (3N8Y) introduces a penalty that significantly decreases the score of the link.

**Table 3:** COX-1 interactions.

Drug ID	Drug name	nAnnoLyze score
**DB00712**	Flurbiprofen	0.97
**DB00328**	Indomethacin	0.97
**DB01600**	Tiaprofenicacid	0.96
**DB00870**	Suprofen	0.96
**DB00821**	Carprofen	0.96
**DB00788**	Naproxen	0.96
**DB00500**	Tolmetin	0.94
**DB00465**	Ketorolac	0.94
**DB00963**	Bromfenac	0.92
**DB00586**	Diclofenac	0.91
**DB06802**	Nepafenac	0.90
**DB01283**	Lumiracoxib	0.90
**DB00784**	Mefenamicacid	0.89
**DB00861**	Diflunisal	0.88
**DB04552**	NiflumicAcid	0.88
**DB00991**	Oxaprozin	0.88
**DB01050**	Ibuprofen	0.87
**DB00939**	Meclofenamicacid	0.86
**DB01399**	Salsalate	0.86
**DB01009**	Ketoprofen	0.86
**DB00605**	Sulindac	0.85

**Table 4:** Aspirin top 10 predicted targets as well as COX-1 and COX-2 scores.

UniProt ID	nAnnoLyze Score	Protein Name
**P07478**	0.94	Trypsin-2
**Q6ZWK6**	0.93	Airway trypsin-like protease 4
**E7ESG9**	0.93	Transmembrane protease serine 4
**A6NL71**	0.92	Transmembrane protease serine 11E
**Q8IXD7**	0.92	Kallikrein-11
**Q0WXX5**	0.92	Kallikrein 11 isoform 1
**A8CED1**	0.92	Trypsin-3
**O60235**	0.92	Transmembrane protease serine 11D protease
**A8CED3**	0.92	Protease serine 3 (mesotrypsin) isoform CRA_c
**A9Z1Y4**	0.92	Protease serine 3
**Q5T7T7**	0.81	COX-1
**A8K802**	0.77	COX-2

**Fig 3:**
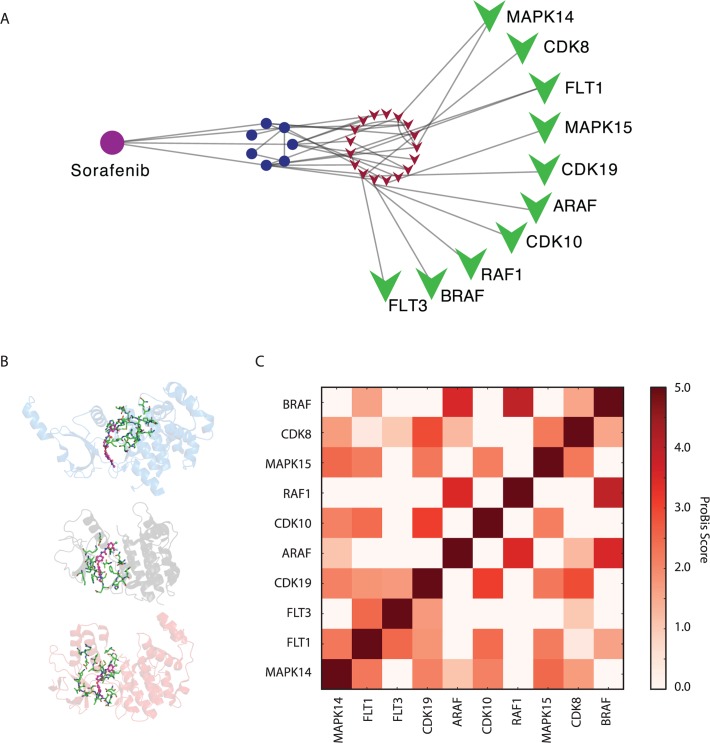
nAnnoLyze multiple-target example. A) Extraction of the Sorafenib sub network. B) Drug and the predicted binding site in CDK8 (PDB 3RGF, blue), BRAF (PDB 1UWH, grey) and MAPK14 (PDB 3GCS, red). C) ProBiS comparison of the binding site of predicted targets for DrugBank molecule Sorafenib (DB00398).

Sorafenib pathway targeting through binding of several proteins. Sorafenib, which is marketed as Nexavar, is an approved drug for the treatment of advanced renal cell carcinoma. It is also in Phase III trials for Hepatocellular carcinoma, Non-small-cell lung carcinoma (NSCLC) and melanoma and in Phase II trials for Myelodysplastic syndrome, Acute Myeloid Leukemia (AML), head and neck, breast, colon, ovarian and pancreatic cancers. Arising as one of the most promising anticancer drugs, Nexavar is known to perform its activity by targeting the Raf/Mek/Erk pathways [36,37]. Specifically it is known to inhibit Raf kinases, Receptor-type tyrosine-protein kinase (FLT3), platelet-derived growth factor (PDGF), Vascular endothelial growth factor receptor 2 & 3 (VEGF2/3) and the Mast/stem cell growth factor receptor Kit. Within our predictions, we found 4 of these links alongside other interesting links for targets involved in the same pathways (Table 5 and Fig. 3A). Interestingly, most of the links have been previously annotated either in DrugBank, PubChem or in the PDB as a crystal structure. However, there are two links not annotated within the predictions, the serine/threonine-protein kinase A-Raf (ARAF) and the Cyclin-dependent kinase 10 (CDK10). ARAF is involved in several pathways, including AML and FoxO signaling and together with FLT3, BRAF, MAPK14 could be a good opportunity to exploit the polyphamarcological profile of Sorafenib against AML. In fact, Phase II trials are showing very promising results in AML combining Sorafenib with other marketed drugs [38,39]. Of the ten predicted targets, only 3 have been co-crystallized with Sorafenib (BRAF, MAPK14 and CDK8), while in the other seven nAnnoLyze proposes the binding site localization of the drug providing insights into the mode of action of the compound. nAnnoLyze predicted the correct binding site for the three targets (Fig. 3B). The predicted binding sites were 75%, 62%, and 86% correct (*i*.*e*., % of predicted residues defined as binding site in LigBase) for CDK8, BRAF, and MAPK14, respectively.

**Table 5:** Sorafenib targets.

Target	Prediction Score	Annotated	Target Structure	KEGG OV Pathways
**MAPK 14 (Q13083)**	0.99	PDB	Yes	MAPK signaling pathway
		PubChem		FoxO signaling pathway
				VEGF signaling pathway
				Rap1 signaling pathway
				RIG-I-like receptor signaling pathway
				Acute myeloid leukemia
**CDK19 (Q9BWU1)**	0.97	PubChem	-	-
**FLT1 (P17948)**	0.90	PubChem	Yes	Ras signaling pathway
				Rap1 signaling pathway
**RAF1 (P04049)**	0.89	DrugBank	Yes	MAPK signaling pathway
		PubChem		Ras signaling pathway
				Rap1 signaling pathway
				VEGF signaling pathway
				FoxO signaling pathway
				Acute myeloid leukemia
**ARAF (Q5H9B3)**	0.88	-	Yes (partially)	FoxO signaling pathway
				Acute myeloid leukemia
**CDK10 (Q15131)**	0.88	-	-	-
**BRAF (Q9Y6T3)**	0.88	DrugBank	Yes	MAPK signaling pathway
		PDB		Rap1 signaling pathway
		PubChem		FoxO signaling pathway
				Acute myeloid leukemia
**CDK8 (P49336)**	0.87	Pubchem	Yes	-
		PDB		
**FLT3 (Q5VTU6)**	0.86	PubChem	Yes	Acute myeloid leukemia
		DrugBank		
**MAPK 15 (Q8TD08)**	0.86	Pubchem	-	-

Since structurally similar binding sites are more likely to bind the same small molecule. We wanted to assess if the 7 predicted binding sites (*i*.*e*., FLT3, CDK10, ARAF, MAPK15, FLT1, RAF1, and CDK19) have similarity with the 3 Sorafenib known binding sites (*i*.*e*., BRAF, MAPK14, and CDK8). All of the 7 predicted binding sites are similar to at least one of the already known (Fig. 3C). Within the annotated interactions with non-crystallized structure, FLT3 is the one with lowest similarity to a known structure (ProBiS Z-score of 1.04 with the CDK8 binding site). Unlike FLT3, FLT1 binding site has MAPK14 as the most similar binding site with a higher score (2.25 ProBiS Z-score). Regarding the Cyclin dependent kinases CDK10 and CDK19 proposed binding sites, CDK10 binding site has a high similarity (ProBiS Z-score of 2.09) with the MAPK14´s one while the CDK19 binding site is almost identical to that of CDK8 (ProBiS 2.9). As expected, RAF predicted protein binding sites ARAF and RAF1 have BRAF binding site as the most similar (3.5 and 3.94 ProBiS Z-scores, respectively). Following the same trend, the MAPK14 binding site is the most similar to MAPK15 (2.51 ProBiS Z-score). Although small changes in the catalytic site could have a dramatic impact on the binding-affinity of a small molecule, the overall high similarity among the Sorafenib predicted binding sites shows a clear trend towards binding site conservation within this set of proteins. This example shows not only the capability of the method to find drug targets but also the possibility to explore pathways rather than individual proteins as targets.

## Discussion

The increase of compound phenotypic screenings over the last years has dramatically increased the number of small molecules with non-annotated protein targets [40–42]. Because target annotation is a crucial step when developing a drug, and specifically the elucidation of the amino acids involved in the interactions is key to understand the mode of action of the compound, many methods have been developed to annotate drug protein targets. However, most of them do not provide any structural information about the link, and for those providing it, the application at proteome scale for any query compound is unfeasible. Here we introduced nAnnoLyze a method for drug target interaction prediction that provides structural details at proteome scale. nAnnoLyze relies on a pre-built network of structural similarities to perform its prediction for any query molecule providing not only the connection between the molecule and its predicted target but also the binding site of the ligand in the protein. It is important to note that nAnnoLyze has been specifically tested for drug-target interaction prediction. The accuracy of our method on less studied compounds, such as non-drug like molecules, could lead to a reduction of the precision and the coverage.

The lack of crystal structure for several proteins in other datasets prompted us to build a new dataset of approved drugs. The reduction of the precision by our previous method [23] with this dataset is indicative of the complexity of the new benchmark. The new dataset includes real set of interactions that better simulates a scenario where the different molecules have different affinities to one or many targets. This addressed a current concern about the possible bias of artificial datasets [33]. Unfortunately, the lack of a real “negative” set of drug-protein pairs (*i*.*e*., pairs of molecules known not to interact) hampered the creation of the complete dataset. To overcome this issue, we generated a set of drug-protein pairs that, so far, are not annotated as interactions. The nAnnoLyze benchmark using these newly created datasets resulted in satisfactory accuracies, especially in light of the fact that the dataset is bound to produce an overestimation of the false-positive rate (*i*.*e*., a drug and a protein are not interacting if they have not been crystallized together) [43]. The limitation of the maximum distance in the search for the shortest pathway can explain some of the missed drug-protein pairs and, consequently, limits the recall reached by the method. Analysis of the precision and recall of specific compounds in the benchmark dataset indicate that nAnnoLyze results in higher accuracy for moderate promiscuous compounds compared to highly promiscuous compounds. Indeed, promiscuous compounds have high-degrees of connectivity in our network, which makes it very difficult to identify specific targets. A similar analysis to identify trends in the accuracy of nAnnoLyze for targets for different protein Pfam families did not result in any clear trend. The usage of binding site to represent a family of targets instead of whole protein domain structures may explain the homogeneity in the performance for different protein families.

Several scores for each prediction permits to explore the effect of the selection of different thresholds values depending of the user needs. For instance, when extracting only the most confident targets for a drug, very low values of Global Z-score will be suitable; while when retrieving the most specific targets for a compound filtering by low values of Local Z-score will be the best option. This, of course, makes it difficult to provide a specific score threshold for the predictions. Despite this, we studied the variation of the performance at different thresholds measured by a ROC curve. The AUC was excellent when using drug names and scores as input feature for the predictions. When only the scores of the predictions were used (that is, treating the compound as anonymous), there was a clear decrease in the AUC suggesting that the method performs better for already known chemical entities rather than for new unseen compounds. This fact makes sense since the method is based upon comparative approaches relating compounds by their structural similarities.

The comparison of the nAnnoLyze method against the original AnnoLyze indicates that our network-based approach predicts drug-protein complexes with higher precision. Importantly, nAnnolyze is a clear progress over Annolyze by improving not only the performance (27-fold higher precision) but also the applicability, since it can be applied to any compound regardless whether it has been previously deposited in the PDB. Moreover, the network-based paradigm implemented in nAnnoLyze allows for the integration of other types of additional information such as the diseases linked to the protein targets, which may eventually allow for drug indication predictions. A successful example of a method for predicting drug-like targets using the modelable human proteome with medical data integration is the Computational Analysis of Novel Drug Opportunities (CANDO) platform [43]. While the aim of our work is accurately predicting drug-protein interactions, future developments of nAnnoLyze could include medical indications of drugs.

To demonstrate the applicability of the method, we screened all the drugs in the DrugBank database against the entire human 3D proteome that could be modeled by comparative protein structure prediction. We not only provided the drug-protein predictions but also the structural binding localization of the interaction. We carefully described two examples of this screening. The first example illustrates the nAnnoLyze ability to correctly (or incorrectly) predict the binding of a NSAIDs set of drugs to the COX-1 human protein. Within the correctly predicted interactions (*i*.*e*., true positives), we included Flurbiprofen and Ibuprofen detailed information about the network routes. In the case of the incorrectly predicted interaction between Aspirin and proteases proteins, the analysis indicates that the clustering in a ligand node of two similar Benzenoids compounds lead to the undesired drug-target association. It is thus likely that adding extra information beyond the chemical similarity during the clustering of the core-network may result in more functionally homogeneous clusters of compounds. Even though, nAnnoLyze was able to reach the two main targets of aspirin (*i*.*e*., COX-1 and COX-2) through alternatives network pathways. However, the lower similarity of the human predicted COX-2 binding site with the ovine COX-1 included in the core network penalized the score of the hit. This example also illustrates the nAnnoLyze capacity of predicting interactions when no crystal structure is available for the target.

The second example studied the polypharmacological profile of the anticancer drug Sorafenib. The method correctly retrieved most of the known targets and proposed some others with structural similarities in the binding site and that are involved in the same metabolic pathways as the known ones. This example shows the possibility of studying pathways rather than individual proteins as drug targets, which could be even more interesting in complex diseases such as cancer or Alzheimer where multiple factors play a role in the progress of the disease.

The major limitation of the method is the restricted applicability because is based on structural data, which is still scarce compared to sequence data. In spite of it, we were able to cover 42% of the human proteome with either a crystal structure or a reliable model. Moreover, the amount of crystal structures in the PDB has significantly increased over the past years [44] and the percentage of a proteome that can be modeled by homology has increased thanks to initiatives like the Protein Structure Initiative [45,46]. The more structural information we have, the more information can be extracted and therefore applied in nAnnoLyze. Indeed, the underlying network in nAnnoLyze can continue growing with the integration of new molecules or sets of biomolecules (both compounds and protein targets). To this end, we have developed a Web server that allows everyone to submit their own sets of compounds and check the predictions against pre-built networks for the human and *Mycobacterium* proteomes. So far, we have applied the method in an open source drug discovery initiative against *Mycobacterium tuberculosis* [31] and are currently working in other projects and initiatives. Our goal is to encourage open source drug discovery by releasing the method with all the predictions expecting that other researchers can benefit from our work. Finally, the scientific community could experimentally validate the predictions providing us a feedback to improve the quality of this tool and of future ones.

## Materials and Methods

**Fig 4:**
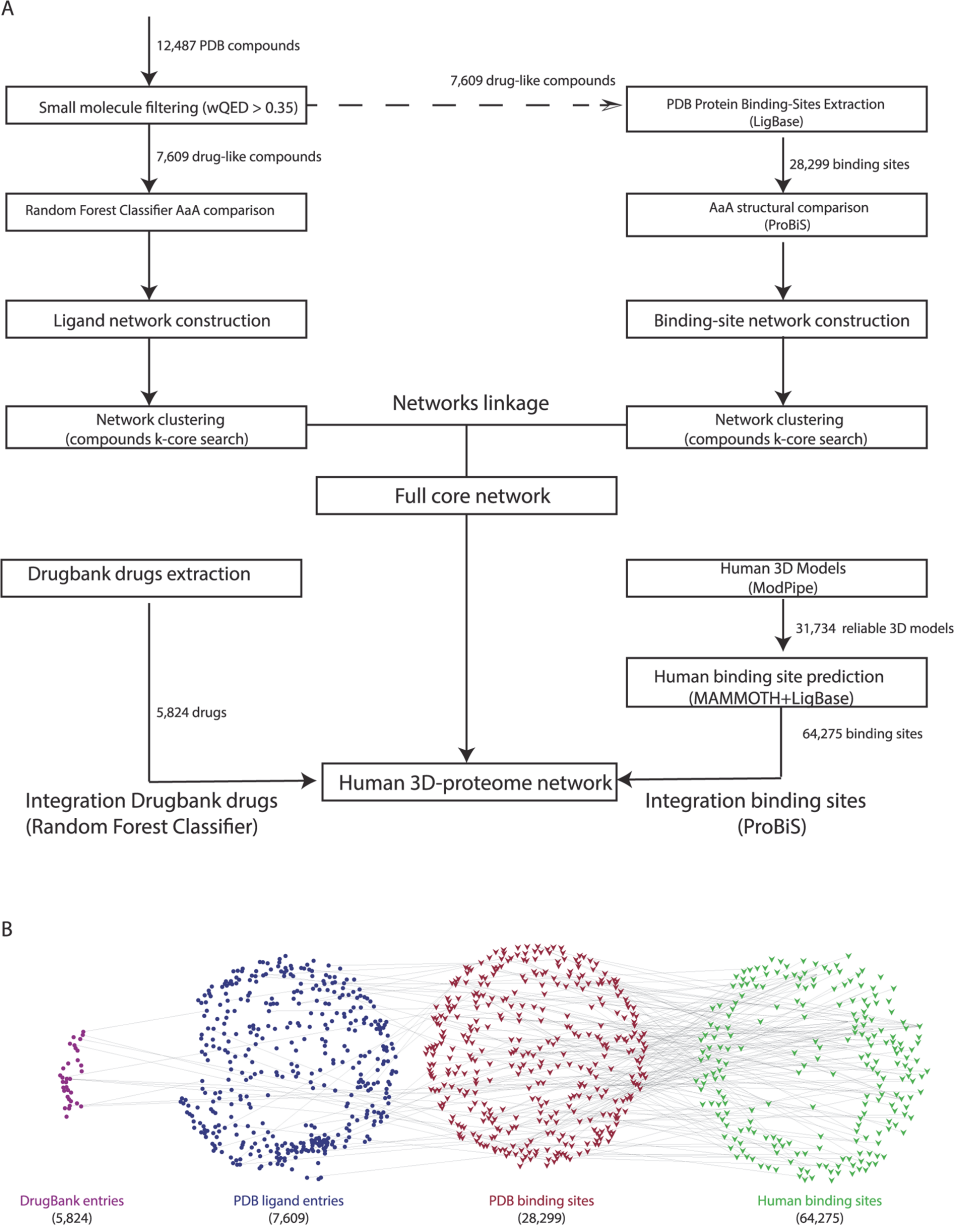
nAnnoLyze network building. A) nAnnoLyze flow chart for building the network of structural similarities between ligands and targets. B) nAnnoLyze underlying sub-networks of drugs (purple circles), compounds in PDB (blue circles), targets in PDB (red triangles), and human target 3D models (green triangles). For easy visualization, the panel shows only part of the final nAnnoLyze network.

Next, we describe the different steps (Fig. 4A) performed to build a bi-partite network of structural similarities and interactions (Fig. 4B). We continue by describing the methods used to assess the accuracy of nAnnoLyze.

### Ligand sub-network

To build the ligand sub-network, only compounds with a pharmaceutical or a biological function on their co-crystallized proteins were retrieved from the PDB. To perform the filtering, we calculated, for each compound in the PDB, the weighted quantitative estimate of drug-likeness (wQED). Briefly, the wQED is calculated by combining a set of the chemical features of the compound (*i*.*e*., molecular weight, octanol-water partition coefficient as LogP, polar surface area, number hydrogen bond donors, number of hydrogen bond acceptors, number of rotatable bonds, number of aromatic rings, and number of possible toxic scaffolds) to quantify its drug-likeness given the pre-calculated value for that chemical features in a gold standard set of drugs [47]. Compounds with good drug-like properties (*i*.*e*., wQED ≥0.35) were selected resulting in 7,609 PDB compounds. Each selected compound was then represented as a vertex in the ligand network. Links between vertices of the network (*i*.*e*., edges) were obtained by structurally comparing all compounds. The weights of the edges were obtained using a Random Forest Classifier (RFC) developed to identify compound similarities [31]. Briefly, the RFC classifier predicts whether two small molecules are likely to bind the same target-binding site by comparing their structural and chemical properties. The usage of a classifier allows for an automatically determination of optimal thresholds after the RFC has been trained with the training-set. Therefore, the all-against-all comparison performed by the RFC resulted in 134,493 pairs of similar compounds. To reduce redundancy in the network we created groups of connected compounds by identifying k-cores in the network. A k-core in a network N, is a maximal connected sub-graph of N in which all vertices have degree at least k. Thus, every k-core in the non-redundant network represents a vertex and edges between vertices indicate the existence of at least one similar compound between the two k-cores. In the ligand network, a k-core would be a set of ligands such every two ligands within the set are similar to each other (*i*.*e*., they have an edge in the network). An edge between two k-cores vertices was given the maximum weight of all possible edges between their constitutive compounds. The resulting non-redundant ligand sub-network had 4,101 vertices connected by 24,856 edges.

### Protein binding sites sub-network

We first downloaded from the LigBase database (February 19^th^, 2013) [48], a database containing all ligand-binding sites of known protein structures, all unique protein binding sites composed of at least seven residues within a radius of 5 Å, binding any of the selected 7,609 highly drug-like compounds in the ligand sub-network. We defined “highly drug-like” compounds as those compounds with very good absorption, distribution, metabolism, and excretion properties (*i*.*e*., with an wQED ≥0.35). This initial protein binding site sub-network resulted in 28,299 binding sites from 22,959 different proteins in the PDB. Next, we populated the network with links (edges) between two proteins by structurally comparing their binding sites. The structural comparison of the binding sites was performed using ProBiS [49], a tool for local structural alignment of binding sites based on geometry as well as physicochemical properties. We defined two binding sites as similar if their similarity Z-score is higher than 2.0. An all-against-all structural comparison of the selected binding sites was performed resulting in 579,155 pairs of similar binding sites. Next, we removed redundancy from the sub-network by applying a similar filtering that is used for the ligand sub-network. The final non-redundant sub-network for binding sites contained 19,487 vertices and 29,811 edges.

### Final bi-partite network

Finally, we joined the two sub-networks by creating edges between protein binding sites and ligands. A binding site was linked to a ligand if both have been experimentally observed to interact (*i*.*e*., a solved structure with the target and the ligand exists in the PDB). The two sub-networks were linked by 22,832 edges and the final nAnnoLyze bi-partite network contained 23,588 vertices and 54,667 edges.

### Integration of the human structural proteome

To populate the nAnnoLyze network with structures for human targets, we downloaded all human 3D models deposited in ModBase (November 11^th^, 2013) [50–52] with at least a 1.1 ModPipe Protein quality score [53]. ModBase is a database of comparative protein structure models calculated by the automatic modeling pipeline ModPipe [53]. The likely accuracy of the ModPipe models is predicted by the ModPipe Protein Quality score defined as a composite score that includes sequence identity to the template, coverage, and the three individual scores: the alignment e-value, z-dope [54], and GA341 [55]. This resulted in a total of 31,734 reliable 3D models from 16,694 unique human target sequences. Next, we structurally compared this set of selected models to any non-redundant (90% sequence identity) set of 29,772 structures from the PDB solved with at least one ligand compound. Structural comparisons between two proteins were performed using the MAMMOTH algorithm, which is based on a fast and accurate heuristic method to find, in a sequence-independent mode, the maximal structural subset between two proteins structures [56]. Four different scores were stored for each structural superposition: percentage of sequence and structure identity for the entire protein and percentage of sequence and structure identity for the residues involved in the binding site of the known structure as defined by LigBase. The structure identity between two structures was defined as the percentage of residues with their Cα atoms within 4 Å after optimal superposition. A binding site in a model was considered then similar to a binding site in a known PDB structure if at least the binding site sequence and structure identity were higher than 40%. This identity cut-off was previously validated in a large-scale comparison of known ligand-protein pairs [23]. A total of 576,675 binding sites were predicted for the human proteins (that is, ∼18 binding sites per model). Due to the high redundancy in the predicted binding sites, we excluded binding sites fulfilling the following requisites: redundant binding sites (*i*.*e*., more than 80% sequence identity to any other binding site) or small binding sites (*i*.*e*., with less than 6 residues). A total of 64,275 binding sites (∼2 binding sites per model) remained after the redundancy and size filtering. Next, we compared all human predicted binding sites against all binding sites in our network using ProBiS resulting in 459,356 similarity links (Z-score > 1.0) between any of the human 64,275 binding sites and the 28,299 binding sites in the network. Every significant pair became an edge with a weight equal to the normalized Z-score of the comparison. The final human network included the 7,609 compounds, the 28,299 known binding sites and the 64,275 human predicted binding sites.

### Integration of the DrugBank compounds

A total of 6,540 small compounds were downloaded from the DrugBank database (May 15^th^, 2013). We then looked for similarity with the compounds present in the PDB ligand sub-network by using our trained RFC classifier as described above. Next, all the drugs were integrated in our network by making an edge between every DrugBank compound and their similar PDB compounds retaining the link with higher RFC when more than one link between a DrugBank compound and one network vertex (*i*.*e*., a k-core of PDB compounds) was found. A total of 5,824 drugs were integrated into the network through 149,538 edges.

### Network-based prediction of DrugBank ligand and human target pairs

Once the network was completed, to predict all possible interactions between DrugBank compounds and any of the modeled targets of the human proteome, we simply calculated the shortest path in the network from every queried DrugBank compound to any human binding sites. We implemented a version of the Dijkstra algorithm that limits the maximum reachable distance in order to speed up the computational time of the search [57]. Each hit was then scored by using the inverse of the sum of all edge weights of the path between the compound and the human target. Such score was then normalized and Z-scored. Specifically, two different Z-scores were calculated for each prediction.

$$Gz=\frac{s-\mu_{G}}{\sigma_{G}}$$

The “Global Z-score” (Gz) is obtained by running the predictions of all drugs present in DrugBank against all targets, obtaining a global mean (*μ*_*G*_) and a global standard deviation (*σ*_*G*_) to Z-score a specific predicted pair. The “Global Z-score” represents how good is a prediction given its score in the constructed network.

$$Lz=\frac{s-\mu_{L}}{\sigma_{L}}$$

The “Local Z-score” (Lz), is similarly calculated by running the predictions of all drugs present in DrugBank retrieving the mean (*μ*_*L*_) and the standard deviation (*σ*_*L*_) of the score for a specific target. The “Local Z-score” represents how good is a prediction for a specific binding site or target. For example, highly promiscuous binding sites tend to have higher local Z-scores.

Finally, we combined the three scores (that is, the inverse of the sum of all edge weights, the global Z-score and the local Z-score through a Random Forest Classifier that aims at predicting the interaction of a compound and a target. Two RFCs were trained with and without the DrugBank ID as an input feature of the compound. The RFC classifier, thus, results in a single Boolean score indicating interaction or non-interaction between the compound and the target. To train the RFC, we used the Weka software for data mining tasks [58].

### nAnnoLyze benchmark

To benchmark nAnnoLyze, we retrieved all the compound-protein complexes for DrugBank approved drugs from the PDB. A total of 213 approved drugs were uniquely mapped into compounds bound to a protein deposited in the PDB. Next, we retrieved all the proteins binding to those compounds resulting in a protein-compound set of 6,282 entries. To test the method, we first created the benchmark network: the 213 compounds were integrated in the clustered network by using the RFC classifier. To avoid overestimation in the benchmark, we did not create any edge between a ligand in the benchmark and any identical (*i*.*e*., RFC score of 1.0) ligand in the network. Next, we extracted from LigBase the 7,074 protein binding sites of the 213 aforementioned compounds and integrated them in the network following the procedure used for the human binding sites. Similarly, we did not create links between identical binding sites in the benchmark and any protein in the network. We then selected all interactions between the 213 compounds and any of the 7,074 binding sites. To assess the accuracy of our method in finding real interactions, we then calculated two different statistics. First, the precision defined as the ratio between the true positives (TP; true drug-protein interactions found by nAnnoLyze) and the sum of TP and false positives (FP, a link between a drug and a protein not in the PDB). Second, the sensitivity (or recall) defined as the ratio of TP and the TP+ false negatives (FN, a link between a compound and protein not found by nAnnoLyze).

### nAnnoLyze Web site implementation

We have implemented a Web server where an end user can retrieve all pre-calculated predictions for the DrugBank and human protein as well as submit its own set of compounds. The server takes as input a compound ID and its SMILE in case of a new compound or only the DrugBank ID in case of a DrugBank drug. Then the user needs to select which organism proteome should be searched against. Currently nAnnoLyze has pre-calculated networks for the human and three *Mycobacterium* proteomes. The server search results in a list of all the predicted compound-protein pairs presented as a sortable table for easy filtering depending on the Global Z-score cut-off. A graphical enrichment of the Gene Ontology Terms [59] and KEGG pathways [60] of the predicted targets is also shown above the result table. Each prediction is further detailed by providing a GLMol based visualization (http://webglmol.sourceforge.jp) of the compound and the protein structure alongside the predicted binding site. All the structural data and all the predictions can be downloaded from the nAnnoLyze Web server at http://www.marciuslab.org/services/nAnnoLyze.
